# 

**Published:** 1892-01-23

**Authors:** 


**Notices of Births, Deaths, and Marriages,** are inserted in this column at a charge of 2s. 6d. for an announcement not exceeding four lines.

### NOTICE TO CORRESPONDENTS.

All MS., Letters, Books for Review, and other matters intended for the Editor should be addressed THE EDITOR, THE LODGE, PORCHESTER SQUARE, LONDON, W.

The Editor cannot undertake to return rejected M.S., even when accompanied by stamped directed envelope.

**Notice to Advertisers and Subscribers.**

All Advertisements, Orders for Copies of THE HOSPITAL, and Business Communications should be addressed to The Publisher, *not to the Editor*, THE HOSPITAL, 140, Strand, W.C.

The Cost of Subscription, post free, is as follows:—Per week, 1½d.; per quarter, 1s. 8d.; per half-year, 3s. 3d.; per annum, 6s. 6d. Foreign:—Per annum, 8s. 8d.; payable, in all cases, in advance.

**"Burdett's Hospital Annual," 1891—1892.**

Third year of publication. 600 pp. Boards, gilt lettering. A most complete guide to British and Colonial Hospitals, Dispensaries, Nursing and Convalescent Institutions, and Asylums. Price 3s. 6d. Published by THE HOSPITAL (Limited), 140, Strand, W.C.

**NOTICE.—The Index for Vol. X. (ending September, 1891), is on sale at the Office of this Journal, price 2½d., post free.**

### GENERAL CHARITIES.

[This Column is devoted to an account of work of charitable institutions other than Medical Charities. The Editor will be glad to receive reports or news of work at 140, Strand. *Philanthropy* should be plainly written on the envelope.]

The Salters' Company have given a donation of fifty guineas to the Irish Distressed Ladies' Fund.

Sir Michael Hicks-Beach will preside at the 94th anniversary of the Royal Masonic Institution for Boys, to be held on June 29th.

Owing to the sad death of the Duke of Clarence the annual meeting of the Whitechapel Working Lads' Institute has been indefinitely postponed. The City Police Festival has also for the present been postponed.

The twenty-first annual meeting of the supporters of **THE CHILDREN'S HOME**, which was started by the Rev. Dr. Stephenson as a refuge with a cottage and two boys, was held last week. The growth of the institution is shown by the fact that at the present time no fewer than 850 boys and girls are being educated, clothed, and taught a trade, at an annual cost of about £18,000.

Miss Frances Ashdown appeals for further help on behalf of **THE CHURCH EXTENSION ASSOCIATION**, 27, Kilburn Park Road, N.W. She does not ask for help for those who will not work, but for those who from age or illness or the slackness of trade are driven into compulsory idleness. It seems superfluous to say that during the cold weather we experience in the winter help is more needed than at any other time of the year. Want of funds cripples the work of the Association at the docks and in Whitechapel, where it is much needed.

The "Old Boys' Supper," which took place last week at the Newport Market Refuge and Industrial School, Coburg Row, Westminster, was an occasion of much delight to the boys. The conditions for admission to the school are that a lad shall be between the ages of eleven and thirteen, free from disease, and medically fit for industrial training, and that his parents shall be unable to provide for him. The majority of the boys, who are given a training as musicians, on leaving the school enlist in regimental bands, and on the occasion of the supper nearly every regiment in the army was represented.

Lord Ashbourne has accepted the office of President of the **BENEVOLENT SOCIETY OF ST. PATRICK** in the room of the Earl of Arran. He will celebrate his accession to this office by presiding at the anniversary festival on March 17. Though the society is an Irish one it is purely non-political, and shillelaghs do not form part of the table decorations or personal adornments at the festival. Much good has been done during the past two centuries by this excellent institution, which at the present time has under its care some four hundred and fifty poor children of Irish or Irish and English extraction.

The Salters' Company have given a donation of ten guineas to the funds of the **AFTER CARE ASSOCIATION FOR POOR AND FRIENDLESS FEMALE CONVALESCENTS** on leaving asylums for the insane, of which association the Earl of Meath is President. The Council urgently appeal for help to carry on and extend the work. Though there are over 240 convalescent homes in this country, we understand that this society is the only one specially designed and available for persons recovering from mental derangement. Cases are assisted by placing patients in convalescent homes, finding suitable work for them, and by giving them grants of money and clothing. Contributions will be received thankfully by the Secretary. Mr. H. Thornhill Roxby, Church House, Dean's Yard, Westminster, S.W.

The Lord Mayor, who appeals to us on behalf of the **MANSION HOUSE COUNCIL ON THE DWELLINGS OF THE POOR**, writes: "During the seven years it has been in existence it has quickened the action of all the local bodies as to sanitary matters, and it is not too much to say that in more than one instance a whole district has been converted from a plague spot and a fever bed into a pleasant neighbourhood of healthy homes. The work of the Council, which is prosecuted by means of voluntary local committees formed of ladies and gentlemen interested in promoting the physical welfare of the poor, is necessarily quiet and unobtrusive, and is apt to be overlooked or forgotten when gifts are being dispensed. I therefore have much pleasure in commending the Council, which is in need of funds now, to prompt and generous support, and shall be happy to receive contributions."

Admiral Sir F. Leopold McClintock asks us to insert the following appeal: What becomes of worn-out Seamen? **THE ROYAL ALFRED AGED MERCHANT SEAMEN'S INSTITUTION**, with an average income of £6,000, provides a home or a pension, as far as means allow. Since its foundation in 1867, it has thus helped 1,040 poor old seamen, who, else, must have ended life in the dreaded workhouse. Two hundred and thirty are now competing for 20 vacancies. These men spend their lives in arduous service at sea, and in trying climates; whereas we, safe at home, while winter tempests rage around us, enjoy at ease the produce of distant lands, the fruit of toil and danger endured by our sailors. On the simplest table stand tea, coffee, sugar, spices; all, together with much more than we consume, purchased by the labour of 360,000 British seamen, too often at the cost of life and limb. What are we doing for our worn-out seamen? Funds, which are very urgently needed, can be sent to the bankers, Williams, Deacon, and Manchester and Salford Bank, or to Mr. W. E. Denny, Secretary, 58, Fenchurch Street, E.C.

### SCRAPS AND GLEANINGS.

THE Hospital Sunday Fund has received an anonymous donation of £500.

THE rating of the Lock Hospital, so excessive as to provoke an appeal, has been reduced from £2,000 to £1,100.

THE Duke of Northumberland has sent eighty pheasants to the Hospital for Epilepsy and Paralysis, Regent's Park.

A BAZAAR, in aid of the Walton Convalescent Home, Bristol, was held in the parish-room of St. Mary, Redcliffe, on the 5th inst.

THE Richmond Convalescent Home, Worthing, which has been temporarily closed will re-open for patients, February 1st.

AT the last meeting of the Metropolitan Asylums Board, it was decided to give Dr. Collie a retiring pension of £210 per annum.

NURSING institutions are being taxed to the utmost just at present. Most of them have to refuse between 40 and 60 applications for nurses daily.

NEW Year's Day was celebrated at Herne Bay by the opening of a Cottage Hospital. The premises are not new, but well adapted to the purpose.

THE Queen has again shown her interest in the London Hospital by sending a present of 110 lb. of cast linen for use in the wards of this large institution.

CHRISTMAS entertainments have been held at the Nottingham General Hospital, Royal Hants County Hospital, and at most similar institutions throughout the Kingdom.

BY command of Her Most Gracious Majesty the Queen, a present of cast linen has been sent to the British Home for Incurables, Clapham Road, from Buckingham Palace.

FRENCH medical papers profess themselves much amused at the notice of a fine of £5 to be inflicted in some parts of England on persons exposing themselves in public places when suffering from influenza.

THE annual report of the Hospital Saturday Fund shows that the receipts for 1891 were £19,683, and expenses £2,819. Receipts were made up as follows: Workshop collection, £14,627; street collections, £4,931; and donations, £144.

A NEW extension to the North Ormesby Cottage Hospital, Middlesborough, was opened by the Countess of Falkland on the 5th inst. Further accommodation was necessary in the out-patients' departments and nurses' quarters.

AN opera, entitled "The Golden Apple," is to be performed by amateurs, at the Royal Albert Hall Theatre, on February 1st, 2nd, and 3rd, at 8 p.m. The proceeds will be given to St. Joseph's Hospital, Kensington Square, and other charities.

A CONCERT in aid of the Royal Free Hospital, Gray's Inn Road, will be held on January 25th, in Prince's Hall, Piccadilly, at 8 o'clock, under the direction of Miss Agnes Larkcom, Madame Helen D'Alton, Miss Agnes Zimmerman, Mr. Dan Price, and many other distinguished artistes will assist.

MESSRS. C. O. ELLISON AND SONS, the architects of the new Toxteth Infirmary, have given an estimate for £28,000, which provides 542 beds. This gives an average expenditure of £53 10s. per bed, which, taking into consideration the high standard of modern requirements in such institutions, is very low.

THE number of distressed Jews in Palestine is very great. Owing to continued snowstorms, the condition of those living in wooden shanties outside Jerusalem is greatly aggravated by the cold. The Society for the Relief of Persecuted Jews is sending out £100 a-week, and Mr. Scott-Moncrieff is at present sheltering and feeding 694.

THE Royal Free Hospital contains 150 beds, and admits about 2,000 sick poor annually. The out-patients number over 24,000. The expenditure amounts to an average of £11, 500, whilst the income from annual subscriptions, &c., reaches £2,500 only. The generous public are therefore especially appealed to to assist so deserving a charity.

LIVERPOOL and Aberdeen have made their Hospital Sunday Collection, and both have suffered from the unhealthy season, which has thinned the congregations in the churches to a marked degree. Some churches in both places have postponed their collection to a more favourable season, and we therefore defer publishing any results until all returns are made.

THE cases of Derby and Toxteth may seem puzzling to the uninitiated. Here is a rate-supported hospital about to be launched into the world at a cost of £60 inclusive per bed. We have also the voluntary hospital proposing to expend £300 per bed. The difference is so startling that we may fairly ask if in the one case too little in the other too much is entertained.

THE active little "bacillus" flourishes in cold climate apparently. At Oxford, where the thermometer registered as low as anywhere in England, he is having a very good time, and so great is the influenza scare that the meeting of the colleges has been postponed for the present. Even the skating grounds, so dear to Oxford residents, present a forlorn appearance.

A CONTEMPORARY gives the following lines, with which we heartily sympathise:

"Long and vainly you have sought us,"
Scoffs the cynical bacillus,
"Now you're certain you have caught us,
Are you sure that you can kill us?"

HIS ROYAL HIGHNESS the late Duke of Clarence and Avondale was the President of the Great Northern Central Hospital, Holloway Road, in which institution he took considerable interest. He presided at a festival dinner which was held at the Hotel Metropole, in May, 1889, in aid of the funds of the hospital which was opened by the Prince and Princess of Wales, in July, 1888, and at the last meeting of the Committee, the Chairman, Mr. C. T. Murdoch, M.P., was deputed to ask him and the Princess Victoria Mary to lay the foundation stone of the additional buildings about to be erected and which will complete the hospital. His loss, therefore, will be severely felt by this charity.

### THE STUDENT OF MEDICINE.

[All communications for this Supplement should be addressed to THE EDITOR of THE HOSPITAL, at 140, Strand, with the words "Students' Column," written in the left hand top corner of the envelope.]

### APPOINTMENTS.

T. Treffry Cockill, M.R.C.S. Eng., L.R.C.P.Lond., appointed House Physician to the North Staffordshire Infirmary and Eye Hospital, vice R. W. Cameron, B.A.Lond., M.R.C.S.Eng., resigned. F. W. Foxcroft, M.B., C.M., appointed Assistant House Surgeon to the General Hospital, Birmingham, vice J. H. Clayton. Frederick W. Foxcroft, M.B., C.M., appointed Assistant House Surgeon to the General Hospital, Birmingham, G. H. Hughes, M.R.C.S., L.R.C.P.Lond., L.S.A.Lond., &c., appointed Junior Resident Medical Officer to the Royal Free Hospital, vice E. Trevethick, M.B.Cantab., resigned. R. Langford Jones, M.R.C.S.Eng., &c., appointed Honorary Physician to the Carnarvonshire and Anglesey Infirmary and Dispensary, vice John Richards, M.R.C.S.Eng., deceased. A. F. Stanley Kent, M.A.Oxen., appointed Demonstrator of Physiology at St.Thomas's Hospital, vice Dr. Copeman. Vincent Moxey, M.R.C.S., L.R.C.P., appointed Junior House Surgeon to the Oldham Infirmary. H. A. Stonham, M.R.C.S., L.R.C.P., appointed Resident Obstetric Assistant Physician to the Westminster Hospital. T. C. Swift, M.R.C.S., appointed Medical Officer to the Foundling Hospital, vice H. W. Statham, resigned. F. W. Ta'Bois, L.D.S.Eng., appointed Dental House Surgeon to Guy's Hospital. Griffith A. Thomas, M.B., C.M.Edin., appointed Medical Officer to the Eye Department of the Oldham Infirmary.

### VACANCIES.

#### Resident Medical Officers.

East London Hospital for Children, Shadwell, E., Medical Officer, unmarried, appointment for two years—Salary £80, with board, &c.

East London Hospital for Children, Shadwell, E., House Surgeon.

General Hospital, Birmingham, Three Assistant House Surgeons.

General Infirmary, Northampton, House Surgeon, not under 23 years of age—Salary £125, with board, &c.

Holloway Sanatorium Hospital for the Insane, Virginia Water, Second Assistant Medical Officer—Salary £200, with board, &c.

Liverpool Dispensaries, Head Surgeon—Salary £200, with board, &c.

London Throat Hospital, 204, Great Portland St., W., House Surgeon.

Norfolk and Norwich Hospital, Norwich, House Surgeon; appointment for six months.

Queen Charlotte's Lying-In Hospital, Marylebone Road, N.W., Medical Officer; appointment for four months—Salary at rate of £60 per annum, with board, &c.

Royal Portsmouth, Portsea, and Gosport Hospital, 137, Queen Street, Portsea, Assistant House Surgeon; appointment for six months—Honorarium of £15 15s., with board, &c.

Royal South Hants Infirmary, Southampton, Assistant House Surgeon.

#### Non-Resident Medical Officers.

Clones Union, Medical Officer to Workhouse—Salary £69.
Dental Hospital of London, Medical Tutor—Salary £40.
General Hospital, Birmingham, Assistant Surgeon—Honorarium £100.

### NOTES FROM THE SCHOOLS.

SIR ANDREW CLARK ON INSTRUCTIONS IN CLINICAL MEDICINE.—An inaugural lecture on the above subject was delivered by Sir Andrew Clark to the students of the London Hospital. The lecturer commenced his address by defining the meaning and explaining the scope of clinical medicine. He pointed out the difficulties met with in the study of this branch of medicine, and discussed the conditions necessary to success in it. Two such conditions were especially insisted on, viz., thoroughness and an undivided attention. Sir Andrew went on to describe the different methods employed in the teaching of clinical medicine. The method he intended to employ was that in which the conditions of teaching resembled the conditions of practice as nearly as possible. The patient, who has not been seen before, is brought before the teacher and students, who investigate the case as if the patient were a private one. The mode of procedure to be adopted was then described in detail. The classes of cases for which this kind of instruction was especially adapted were the slighter forms of chronic disease, and functional diseases, for the proper teaching of which no adequate provision was made in our general hospitals. Sir Andrew, in conclusion, urged the importance of a proper regard and reasonable consideration on the part of the examiner for the susceptibilities and feelings of the patients who were being examined, and who were voluntarily undergoing a considerable ordeal.

### THE STUDENT OF MEDICINE—(*continued*).

### PASS LISTS.

### University of London.

M.S. EXAMINATION.

R. C. Bailey (gold medal), and R. Bird, M.D.

B.S. EXAMINATION.

FIRST DIVISION.—G. F. Blacker, E. E. Blomfield, G. S. Buchanan, B.Sc., H. G. G. Cook. T. B. P. Davies, D. Drew, W. McA. Eccles, R. H. Elliot, H. Hodgson, B. W. Hogarth, R. Pickard, J. E. Platt, M.D., A. T. Rake, W. G. Rogers, H. J. Waring, B.Sc., and A. S. Wohlmann.

SECOND DIVISION.—S. Bueno de Mesquita, T. P. Cowen, H. J. Curtis, D. R. Green, Alice J. McLaren. H. M. Richards, J. L. Roberts, M.D., B.A., B.Sc., C. E. Salter, G. A. Simmons. C. R. Stevens. F. R. P. Taylor A. E. Tebb, A. Thomas, E. E. Ware, J. G. Wilson, and J. Young.

The following gentlemen have passed the examination for Honours at the recent B S. examination :—

FIRST CLASS.—G. F. Blacker (Scholarship and Gold Medal), University College; H. G. G. Cook (Gold Medal), St. Bartholomew's Hospital; J. L. Roberts, M.D., B.A., B.Sc., Guy's Hospital; R. Pickard, St. Bartholomew's Hospital, and W. G. Rogers, Guy's Hospital (equal).

SECOND CLASS.—W. McAdam Eccles, St. Bartholomew's Hospital; T. B. P. Davies, Guy's Hospital; R. H. Elliot and H. J. Waring, B.Sc., St. Bartholomew's Hospital (equal); T. P. Cowen, St. Thomas's Hospital, and H. H. Hodgson, Guy's Hospital (equal).

THIRD CLASS.—J. E. Platt, M.D., Owen's College and Manchester Royal Infirmary; A. T. Rake and A. S. Wohlmann, Guy's Hospital (equal); H. M. Richards, University College.

### Society of Apothecaries of London.

The following candidates have passed the examination in the subjects indicated :—

SURGERY.—C. W. Allen, J. Dulberg, C. M. Fleury, E. E. Frazer, W. A. Haslam, S. P. Hopewell, C. J. Kelly, J. T. Mills, Walker Overend, Wilkinson Overend, T. E. Pallett. W. M. Palmer, H. C. Powell, V. J. R. Robin, J. A. Samuel, C. H. C. Visick, W. A. Ward, S. Wimbush, A L. Wykham, A. T. Wysard.

MEDICINE. FORENSIC MEDICINE, AND MIDWIFERY.—A. D. Bensusan, D. J. Caddy, C. M. Fleury, H. J. Frederick. W. H. Goodson, A. Graydon, R. R. Hatherell, W. Hawker, C. J. Kelly, J. E. Ramsay, A. T. Wysard.

MEDICINE AND FORENSIC MEDICINE.—J. Dulberg, W. M. Palmer.

MEDICINE AND MIDWIFERY.—W. A. Haslam.

FORENSIC MEDICINE AND MIDWIFERY.—C. S. Lewis.

FORENSIC MEDICINE.—H. J. Foster, P. G. Laver, H. C. Powell, T. W. Smith.

To Messrs. Allen, Fleury, Goodson, Graydon, Hatherell, Hawker, Kelly, Laver, Overend (Wilkinson), Powell, Ramsay, Samuel, Visick, Wykham, and Wysard was granted the Diploma of the Society, entitling them to practice Medicine, Surgery, and Midwifery; enabling the holder to compete for Medical Appointments in the Army, Navy, and India Service, also for Poor-law appointments.

EXAMINATION IN ARTS.

The following have passed in all the subjects required for registration :—

FIRST CLASS (in order of merit).—F. C. Blakiston, E. S. Crispin, J. C. Hollocombe, B. H. E. Evans.

SECOND CLASS (in alphabetical order).—M. C. B. Anderson, H. L. Apthorp, A. Atherton, C. E. Carter, M. S. Coghill, G. E. Duncan, W. R. Field, A. H. Gadsden, J. P. E. Henery, B. Hogan, J. B. B. Howatt, T. W. Hyde, E. Joseph, L. F. Leslie, H. R. Marsh, H. R. Miller, J. Milligan, P. D. Pywell, A. L. Rozelaar, F. S. Vine, H. J. Walker, F. E. Wayte, E. M. Wells, R. G. Whiting.

PRIMARY EXAMINATION, PART I.—JANUARY, 1892.

The following candidates passed in :

CHEMISTRY, MATERIA MEDICA, BOTANY, AND PHARMACY.—A. W. Fyffe, Galway and Belfast; J. Scarr, Littleborough; G. H. Wilkinson, Queen's College, Birmingham.

CHEMISTRY.—L. M. Blake, Royal Free Hospital.

MATERIA MEDICA, BOTANY, AND PHARMACY.—A. Hilton, Manchester, Owen's College; A. W. Taylor, Edinburgh University; E. Wright, Queen's College, Birmingham.

MATERIA MEDICA AND BOTANY.—F. T. Cole, King's College.

PHARMACY.—E. C. Bond and E. E. Evans, Royal Free Hospital; F. Mannin, Dublin.

PRIMARY EXAMINATION, PART II.

The following candidates passed in :

ANATOMY AND PHYSIOLOGY.—R. R. P. S. Bowker, Middlesex Hospital; W. J. H. Dawson, St. Thomas's Hospital; G. H. Pooley, Cambridge University and St. George's Hospital; G. H. Proctor, London Hospital; S. Smith, Middlesex Hospital; W. E. Stanton, Cambridge University and London Hospital; E. P. Staples, Cambridge University and St. Mary's Hospital; F. W. Sell, Cambridge University; F. A. Storr, Leeds, Yorkshire College; S. A. Stride, London Hospital; A. W. Taylor, Edinburgh; J. Thornton. London Hospital.

ANATOMY.—S. Foster, St. Bartholomew's Hospital; A. E. Freer, St. Mary's Hospital; E. Grange, Edinburgh University; F. R. Heap, Bristol; T. Hopps, Manchester, Owens College; F. H. Humphris, Edinburgh University; T. E. Johnson and J. Joule, London Hospital; G. W.

### THE STUDENT OF MEDICINE—(*continued*).

Pauli, Bristol; A. J. Pollard, Leeds, Yorkshire College; C. J. Underwood, Charing Cross Hospital and London Hospital; A. Whitby, Dublin.

PHYSIOLOGY.—R. K. Bayley, St. Mary's Hospital; E. T. Fitzpatrick, Dublin; T. Gregg, St. Bartholomew's Hospital; F. B. Hargreaves, Manchester, Owens College; J. A. F. Hatch, Dublin; A. Hilton, Manchester, Owens College; E. S. L. Lovell, Charing Cross Hospital; A. C. McLean, King's College; J. B. D. St. Cyr, St. Bartholomew's Hospital; J. J. Winn, London Hospital.

### Royal College of Surgeons, Edinburgh.

The following gentlemen have been admitted Fellows of the College since last publication: H. W. F. Powell, L.F.P. and S.G.; W. L. Woollcombe, M.R.C.S.Eng.; C. Seal, M.R.C.S.Eng.; A. L. Turner, M.B., C.M; E. J. Gibson-Berkley, M.R.C.S.Eng.; A. M. Gledden, M.R.C.S.Eng.; D. C. Longden, M.B., C.M.; A. Taylor, L.R.C.P.Irel.; P. C. E. D'Erf Wheeler, M.R.C.S.Eng.; and G. A. Hawkins-Ambler, L.R.C.P.Irel.

The following gentlemen have been admitted Fellows without examination: I. Mossop, L.R.C.P. and S.E.; and D. Little, M.D.

The following gentlemen were admitted Licentiates of the College: J. W. Goldon, J. Fuller, A. E. Dyas, E. B. Fuller, W. T. Crawford, C. J. B. Johnson, and J. H. Thompson.

The following gentlemen received the Diploma in Public Health of the College: C. G. R. Scott, M.R.C.S.Eng., and R. Beveridge, M.B., C.M.

The annual examination in Materia Medica and Therapeutics for the gold medal presented to the College by Colonel William Lorimer Bathgate, in memory of his late father, Mr. William McPhune, Bathgate, F.R.C.S.E., took place on October 31st. Five candidates underwent examination, and the medal has been awarded to Mr. Patrick McElwaine.

### University of Oxford.

In a congregation held on the 17th ult. the degree of D.Med. was conferred on H. J. Tylden, Exeter.

### Faculty of Physicians and Surgeons of Glasgow.

At the December Examination in Public Health the following gentlemen were admitted Diplomates in Public Health: J. Alexander, M.D., Wick; F. H. May, L.R.C.P., Birmingham.

### University of Cambridge.

At the Third Examination for Medical and Surgical degrees the following gentlemen were examined and approved:—

PART I.—Ds. Barrett, Cai.; Ds. Brooksbank, Trin. H.; Ds. Carr, Trin.; Ds. Chappel, Cai.; Ds. Connon, Cai.; Ds. Coulby, Trin.; Ds. Crofton-Atkins, Clare; Ds. Doman, Cai; Mag. Durham, King's; Ds. Evans, Joh.; Ds. Floyd, Clare; Ds. Heppell, Cai.; Ds. Hollis, Sid.; Ds. Joyce, Queen's; Ds. Langdon-Down, P., Trin.; Ds. Lister, W. T., Trin.; Ds. Mason, G. A., Joh.; Ds Master, H. Cav.; Mag. Melsome, Queen's; Ds. Paterson, Trin.; Pinder, Queen's; Ds. Thomas, C. N., Trin.; Ds. Thomas, H. Cav.; Ds. Walker, W. W., Trin.; Ds. Wilson, A. H., Christ's; Ds. Windsor, Emman; Ds. Young, Joh.

PART II.—Ds. Attfield, Pet.; Ds. Atlee, J., Joh.; Ds. Bolus, Jesus; Ds. Bradford, Cai.; Carling, W.; Ds. Cowell, Joh.; Ds. Craig, Cai.; Ds. Dorman, Clare; Ds. Drake, Clare; Mag. Durham, King's; Mag. Eccles, Down; Ds. Edmondson, Joh.; Ds. Evans, Joh.; Ds. Ferguson, Cai; Ds. Fisher, Cai.; Ds. Fooks, Jesus; Ds. Freer, Cai.; Ds. Gillam, H. Cav.; Mag. Gillibrand, Queen's; Ds. Glover, L G., Joh.; Ds. Godson, A. H., Joh.; Ds. Grimsdale, Cai.; Ds. Hulbert, Trin.; Ds. Kent, Trin.; Ds. Law, Christ's; Ds. Lee, H. Cav.; Ds. Mason, G. A., Joh.; Ds. Mercer, W. B., Cai.; Ds. Perkins, Emman.; Ds. Pridham, Cai.; Ds. Senior, Queen's; Ds. Shaw, W. H. C., Jesus; Ds. Simpson, H., Joh.; Ds. Stack, E. H. E., Pemb.; Mag. Stubbs, Trin.; Ds. Turner, F. M., Trin.; Mag. Wallace; Wrench, H. Cav.

### London Hospital.

Session 1890-91.—Entrance Science Scholarships, instituted 1875, given for proficiency in the subjects required for the preliminary scientific M.B examination at the University of London—£60 scholarship, Mr. M. Cameron; £40 scholarship, Mr. J. Haverson. Buxton Scholarships, given for proficiency in the subjects required for the preliminary examinations—£30 scholarship, Mr. W. G. Mortimer; £20 scholarship, Mr. E. H. Cooper. Clinical Medicine—£20 scholarship given jointly by the House Committee and the Medical Council, Mr. J. Mulvany; honorary certificates, Mr. E. E. Blomfield and Mr. J. M'Neal. Clinical Surgery—£20 scholarship, given jointly by the House Committee and the Medical Council, Mr. J. Mulvany; honorary certificate, Mr. J. M'Neal. Clinical Obstetrics—£20 scholarship, given jointly by the House Committee and the Medical Council, Mr. E. E. Blomfield; honorary certificate, Mr. J. Mulvany. Duckworth Nelson Prize—£10, for practical medicine and surgery, Mr. J. Mulvany; honorary certificate, Mr. J. M'Neal. Letheby Prize—£30 prize for proficiency in chemistry, Mr. H. L. Barnard; honorary certificate, Mr. W. S. H. Sequiera. Hutchinson Prize—£35, essay on Clinical Surgery, Mr. J. Thomas. Anatomy, Physiology, and Chemistry—£25 Scholarship, given by the Medical Council, G. H. Cowen; honorary certificate, Mr. C. P. Harris. Anatomy and Physiology—£20 Scholarship, given by the Medical Council, Mr. W. B. Butler; honorary certificates, Mr. M. Cameron and Mr. H. W. Grattan. Dressers' Prizes for Zeal, Efficiency, and Knowledge of Minor Surgery, given by the House Committee—£15 prizes, Mr. D. W. F. Latham and Mr. J. E. Gilbert Rogers; £10 prize, Mr. V. J. Batteson; £5 prizes, Mr. A. E. Kennedy and Mr. L. A. Smith. Practical Anatomy Prizes—£6 prize, Mr. C. P. Harris; £4 prize, Mr. A. W. Riley; £3 prize, Mr. A. D. Howard.

